# Manipulating NK cellular therapy from cancer to invasive fungal infection: promises and challenges

**DOI:** 10.3389/fimmu.2022.1044946

**Published:** 2023-01-11

**Authors:** Bernice Ling Zhi Oh, Louis Wei Yong Chan, Louis Yi Ann Chai

**Affiliations:** ^1^ VIVA-University Children’s Cancer Centre, Khoo-Teck Puat-National University Children’s Medical Institute, National University Hospital, Singapore, Singapore; ^2^ Department of Paediatrics, Yong Loo Lin School of Medicine, National University Singapore, Singapore, Singapore; ^3^ Clinician Scientist Academy, National University Health System, Singapore, Singapore; ^4^ Division of Infectious Diseases, Department of Medicine, National University Health System, Singapore, Singapore; ^5^ Department of Medicine, Yong Loo Lin School of Medicine, National University of Singapore, Singapore, Singapore; ^6^ Synthetic Biology for Clinical and Technological Innovation (SynCTI), National University of Singapore, Singapore, Singapore

**Keywords:** augmentation, cytokines, chimeric antigen receptor, adoptive cellular therapy, innate immunity

## Abstract

The ideal strategy to fight an infection involves both (i) weakening the invading pathogen through conventional antimicrobial therapy, and (ii) strengthening defense through the augmentation of host immunity. This is even more pertinent in the context of invasive fungal infections whereby the majority of patients have altered immunity and are unable to mount an appropriate host response against the pathogen. Natural killer (NK) cells fit the requirement of an efficient, innate executioner of both tumour cells and pathogens – their unique, targeted cell killing mechanism, combined with other arms of the immune system, make them potent effectors. These characteristics, together with their ready availability (given the various sources of extrinsic NK cells available for harvesting), make NK cells an attractive choice as adoptive cellular therapy against fungi in invasive infections. Improved techniques in *ex vivo* NK cell activation with expansion, and more importantly, recent advances in genetic engineering including state-of-the-art chimeric antigen receptor platform development, have presented an opportune moment to harness this novel therapeutic as a key component of a multipronged strategy against invasive fungal infections.

## Introduction

Despite the availability of treatment options, the risk of mortality from invasive fungal infections (IFIs) remain high, above 50% particularly for mould infections. While this may be attributable in part to the limited range of effective anti-fungal treatments available, the dismal outcomes are primarily accounted for by susceptible, at-risk cohorts such as the severely immunocompromised recipients of chemotherapy, stem cell or organ transplants, as well as those receiving immunosuppressive biologic therapy. These patients may not be competent in mounting an effective host response against the pathogen, even with the aid of an appropriate antimicrobial. On this background, the augmentation of host immunity and antimicrobial responses through the replacement of immune cellular components is a logical strategy. Advances in recent decades have cast a spotlight on T lymphocytes and natural killer (NK) cells which have emerged as promising adoptive cellular therapeutic modalities. This review focuses on capitalizing NK cellular therapy against IFIs, taking reference from parallels in oncology and T cell immunotherapy.

## Biology of NK cells

Large lymphocytes with granular cytoplasm by morphology, NK cells are innate lymphoid cells that circulate in the blood, bone marrow and tissue (1). A distinct feature is the expression of CD56 [also known as neural cell adhesion molecule 1 (NCAM1)] and the absence of T cell receptors (2). NK cells are derived from hematopoietic progenitors in the bone marrow where they undergo maturation; NK cell development also occurs in other lymphoid organs (3). However, unlike T cells, trafficking to the thymus is not a requisite for NK cell maturation (4).

NK cells have traditionally been classified as either immunomodulatory (CD56brightCD16dim/negative) or cytotoxic (CD56dimCD16bright), based on CD56 and CD16 expression. More recently, however, advances in multi-parameter cytometry and single cell proteogenomics have led to discoveries of more diverse populations of NK cells. NK cells have been shown to possess memory-like function through higher intensity recall responses following repeated exposures (5, 6). For example, in c*ytomegalovirus* (CMV) infections (7–9), studies have shown that a higher proportion of NK cells demonstrate increased expression of NKG2C, an activating receptor which recognizes and binds to CMV peptide (10).

Peripheral homeostatic maintenance allows NK cells to persist in the blood even when differentiation of progenitor cells fails (11, 12). Human NK cell turnover in the blood occurs every 2 weeks and *in vivo* doubling time is estimated to be 13.5 days (13, 14). Continuously stimulated NK cells are able to achieve a median of 16 doublings before immune senescence. Reduction of telomere shortening through overexpression of telomerase reverse transcriptase (TERT) increases NK cell lifespan by allowing additional doublings (14).

## Role of NK cells in host immunity

The function of NK cells in the immune system is twofold: first, as effector cells in the control of both microbial infections and cancer; second, as regulatory cells that interact with other immune cells such as T cells, B cells and dendritic cells (15).

NK cells play a pivotal role in the control of microbial infections in the immune system. When pathogens reduce the expression of HLA-I on infected cells to evade T cell surveillance, NK cells activate themselves in response to the low HLA-I expression (16, 17). To kill microbe-infected cells, NK cells mediate antibody-dependent cell cytotoxicity (ADCC) on antibody-bound cells (18); in addition, NK cells bind to death receptors such as Fas ligand and tumour necrosis factor-related apoptosis-inducing ligand (TRAIL) to induce apoptosis and cytotoxicity (19). NK cells also augment host anti-microbial responses through the secretion of proinflammatory cytokines including interferon-gamma (IFNγ) (20). NK cells can mount adaptive responses such as the generation of specific NK cells with memory function following repeated exposure, given that different microbes evoke distinct NK cell mediated responses (10, 21, 22).

NK cells further serve as regulators of the immune response through various positive and negative feedback loops, as well as through direct effects on other immune cells (23). NK cells can also interact with dendritic cells by cross presenting antigens of killed targets, triggering a cascade of downstream responses mediated by dendritic cells (24, 25). The secreted cytokines, IFNγ and tumour necrosis factor alpha (TNFα), also support the maturation of dendritic cells which secrete IL-12, which further enhances NK cell activity (26, 27). NK cells can also act directly on T and B cells to moderate overly activated responses against pathogens (15, 28).

Analogously, in cancer, NK cells mediate cytotoxicity against tumour cells through direct interactions based on the presence of NKG2D ligands (which are upregulated in sarcomas) or absence of MHC class I expression (such as in lymphomas) of NK cell receptor ligands on cancer cells (29–31). Cytotoxicity can also be mediated through ADCC as they express constant region (Fc) receptor CD16 which allows recognition and binding to IgG1 and IgG3 antibodies coated on cancer cells. NK cells have also been implicated in tumour immunosurveillance – this is most evident in patients with primary NK cell immunodeficiencies who have been observed to have a higher risk of developing malignancies (32–34). Longitudinal population-based studies suggest a higher relative risk of cancer in those with lower NK cell cytotoxicity (35). Additionally, reduced NK cell function and lower NK cell infiltration in tumours have also been linked to poorer outcomes in cancer patients (36). Conversely, increased NK cell numbers and function were associated with treatment response and disease control in haematological malignancies (37–39).

## Licensing and activation

### Licensing

Licensing refers to the acquisition of functional competency during NK cell maturation that mediates the capacity of NK cells to respond to activating signals. This is dependent on interactions between killer cell immunoglobulin-like receptors (KIRs) and major histocompatibility complex (MHC) molecules that belong to self (40, 41). MHC class I molecules bind to KIRs which downregulate NK cell function through immunoreceptor tyrosine-based inhibitory motifs (ITIMs) to dampen signals through tyrosine phosphatases (42); these then minimize the destruction of healthy cells (43). NK cell functionality is determined by the amount and type of MHC class I alleles in that interaction with KIRs (42). Once NK cells are functionally competent, mature NK cells are suppressed by ligation of intact self-MHC; suppression is lifted if MHC is altered or reduced (42). The responsiveness of NK cells can be reset or altered in an environment with variant MHC expression, while unlicensed NK cells can still stimulate adaptive immune responses (44, 45). In addition, KIR-mediated inhibition of mature NK cells can also be overcome by stronger activating stimuli (46).

### Activation

Activating receptors transmit signals *via* various pathways. The natural NK cytotoxicity receptors, NKp30 and NKp46, operate through the immunoreceptor tyrosine-based activation motifs (ITAMs) of high-affinity IgE receptor (FcεRIγ) and CD3ζ, while NKp44, CD94-NKG2C, KIR2DS1, KIR2DS2, KIR2DS4 and KIR3DS1 operate *via* the ITAMs bearing DAP12 adaptor (47). NK cell activation also depends on co-stimulation by molecules such as [1] DNAM1, using a tyrosine and asparagine-based motif (48), [2] 2B4, signalling through an immunoreceptor tyrosine-based switch motif (ITSM) with NK cell receptor 2B4 (49); and, [3] OX40, recruiting tumour necrosis factor-associated factors to initiate signals (50). Signals from activating receptors could be countered by inhibitory receptors (43, 51, 52). The heterodimer CD94-NKG2A engages HLA-E and transduces inhibitory signals *via* ITIMs (53, 54); this counteracts the activating signals of CD94-NKG2C which then binds to HLA-E with lower affinity (55). The ligands of activating co-stimulatory molecules such as DNAM1 can also bind to inhibitory T cell immunoreceptor with immunoglobulin and ITIM domains (TIGIT) and CD96 (16). NK activation is also mediated by antibodies binding to CD16 Fc receptor, which induces phosphorylation of ITAM domains of FcεRIγ and CD3ζ - launching a cascade that culminates in cytotoxicity of the target cells known as ADCC (56, 57).

## Regulatory cytokines

Cytokines are crucial for activation of NK cells, especially in the context of naïve NK cells where binding to individual activating receptors is generally insufficient to trigger a cytotoxic response (58). Interleukin 2 (IL-2) and interleukin 15 (IL-15) are the most well described and commonly used cytokines which can activate NK cells.

Given that the IL-15 receptor (IL15R) shares signalling β and γ subunits with the IL-2 receptor (59), both IL-2 and IL-15 enhance signalling from activating receptors of NK cells (58, 60). In addition, IL-15 also promotes their survival and proliferation (59, 61, 62). Produced primarily by monocytes, macrophages and dendritic cells, IL-15 forms complexes with the α-chain of IL15R on cell surfaces (63, 64). IL-15 demonstrates superior function when presented on the cell surface as membrane-bound IL-15 (mbIL-15) as compared to its soluble form (64). Transduction of mbIL-15 on human NK cells results in increased longer-term survival compared to its secreted form (65). This is due to the paradoxical effect of IL-15 which induces cytokine-inducible SH2-containing protein (CIS) expression, which dampens NK cell activation by promoting the degradation of the tyrosine kinase JAK1 (66).

Other cytokines such as IL-12, IL-18 and IL-21 have also been used in varying combinations to increase the cytotoxicity of NK cells (67, 68). Specifically, human NK cells cultured for 16 hours with IL-12, IL-15 and IL-18, then washed and maintained in IL-15 for 1-2 weeks, exhibited a “memory-like” form upon re-stimulation (69). The complex interplay between the various cytokines may lead to other effects as well. For example, IL-12 can induce expression of inhibitory receptor NKG2A (69, 70) and IL-21 may inhibit IL-15 induced NK cell expansion (71).

## Cytotoxic effector capabilities of NK cells

NK cell-mediated cell killing occurs through activation triggered by the formation of a synapse with the target cell. Lytic granules transported on microtubules then concentrate on the synapse, where they fuse with the plasma membrane and are then released (72). Perforin, one of the main components of these granules, forms pores in the cells, leading to osmotic lysis (73). Granzymes, another component of these granules, enter the cells through the pores created by perforin and trigger apoptosis of the cells by activating caspases (72). Lytic granules are very potent as a solitary units in killing target cells (73). NK cells can also proceed to kill other target cells even after degranulation (74); as they can also kill cells through the expression of FAS ligand and TNF-related apoptosis -inducing ligand (TRAIL) (74). In addition to direct cytotoxicity, NK cells also attract and stimulate other immune cells such as dendritic cells (75) through the secretion of various cytokines, chemokines and growth factors (43). An example would be the cytokine IFNγ, which promotes polarization of T helper 1 cells, induces MHC class II expression on antigen presenting cells and, activates macrophages (76).

## Specific cytotoxic capabilities of NK cells against fungi

Against fungi, the modus operandi of NK cells is similar: direct killing either *via* degranulation releasing perforin or granzyme, or secretion of effector cytokines (such as IFNγ and TNFα); where both induce phagocytosis and result in cell death. *In vitro*, NK cells have exhibited phagocytic-like characteristics, engulfing *Candida* yeast cells in a contact-dependent manner (77). NK cells execute their main effector role by means of direct cytolytic capacity against fungi, through the release of lytic molecules pre-loaded in granules within the NK cell cytoplasm. Perforin causes direct pore formation in the fungal cell membrane disrupting membrane integrity and facilitating entry of granzymes into the pathogen. Both perforin and granzymes synergize to mediate apoptosis of target cells. Granzymes, classically represented by Granzyme B, induces apoptotic target cell death through caspase-dependent pathways as well as release of inflammatory cytokines such as interleukin 1 alpha and beta (78). The anti-fungal role played by perforins has been well demonstrated against *Rhizopus* (79), *Aspergillus* (80) and *Candida* (77). NK-directed killing is also morphotype-specific, for example, in the case of *Aspergillus* or *Rhizopus*, germinating conidia or hyphae are the targets of NK cells.

Host recognition of fungi involves classic pathogen recognition receptors such as toll like receptor (TLR) 2 (81, 82) and C type lectin receptor Dectin-1 (also known as CLEC 7a) (83); which recognize beta-glucan in the fungal cell wall (81). NK-specific receptors, such as Natural Killer Cell Receptors (NCR) subtypes NKp30 and NKp46, have also been implicated. Against *Candida* and *Cryptococcus*, NKp30 receptors have been shown to mediate perforin release and fungal cytotoxicity through the PI3K-ERK1/2 pathway (84, 85). Binding between the *Candida* cell wall adhesins Epa1, Epa6, and Epa7, and NKp46 receptor and its mouse orthologue NCR1, resulted in enhanced NK activation through degranulation marker CD107, resulting in reduced fungal burden (86). Most recently, the *Candida* adhesin Agglutinin-Like Sequences (ALS) was identified as a ligand for TIGIT, an inhibitory checkpoint inhibitor on NK cells (87). CD56, a classical NK cell marker, has also been shown to be a major pathogen recognition receptor which directly interacts with *A. fumigatus*. Blockade of CD56 diminished NK activation, with reduced inflammatory cytokine secretion against *A. fumigatus* (88). ‘Death receptors’ TRAIL-R and Fas by TRAIL and Fas ligand (FasL) (CD95L) have also been observed to be triggered during the degranulation process (18).

IFNγ and proinflammatory TNFα are pivotal mediators of type 1 T helper cell (Th1) host response against fungi (89), while granulocyte-macrophage colony stimulating factor (GMCSF) activates the obligate phagocytes against fungi, macrophages (77). The major source of secreted IFNγ against fungi originates from NK cells. IFNγ is central in orchestrating the host immune response against the pathogen, serving as a bridge between the innate and adaptive immune systems. IFNγ drives the differentiation of CD4 cells towards Th1 subsets on its own (90), or in synergy with TNFα (91, 92) and also mediates NK cross talk with dendritic cells (81). IFNγ also activates tissue macrophages, as shown in the context of *Cryptococcus* infections (93, 94). Against *Candida*, IFNγ has also been shown to augment the potency of polymorphonuclear neutrophils (77). Furthermore, NK cells also secrete chemokines such as CXCR2, CCL3/MIP-1α, CCL4/MIP-1β and CCL5/RANTES. The production of these chemokines may be titrated on a dose-dependent response, as seen in *Aspergillus* infections (95). These chemokines serve to fine-tune NK cell migration, cytotoxicity, and neutrophilic anti-fungal activity (96, 97).

## Development and refinement of NK cellular anti-fungal treatment: *in-vitro* and *in-vivo* studies

The earliest attempts at utilizing NK cells of murine origin against fungi began more than 30 years ago (**Table 1**). The process of purification involved passing murine splenocytes through nylon wool, retaining monocytes and B cells. The non-adherent fraction was further differentiated through Percoll gradient fractionation, or selected by panning and rosetting with sheep erythrocytes. The NK-containing cellular fractions demonstrated anti-fungal effects, when tested *in vitro* with co-incubation against pathogenic fungi of interest, *Cryptococcus* and *Paracoccidioides* (98–100). Growth inhibition in excess of 50% could be achieved, albeit with higher effector-to-target (E:T) ratios. The first attempts with human peripheral blood lymphocytes followed on, through density gradient centrifugation and selection for Leu-11b directed against NK surface Fc receptors. *In vitro*, co-incubation setting against *Coccidioides*, human NK-enriched cells inhibited *C. immitis* endospores and spherules effectively. In this setting, interferon was used for immune augmentation and was shown to effectively enhance leucocyte-mediated fungal killing (101). As IL-2 appeared to enhance NK cell activation (102), this was added to CD16/CD56+ cell fractions against *Cryptococcus* with demonstrable efficacy (103). 

**Table 1:** Progressive development of fungal-specific NK cellular platforms.

Type	Fungi type	Cell type or platform	NK cell source	Method of NK cell preparation	MOI Effector-to-Target (E:T) or cell dose; Exposure time	Priming / Stimulation	Immuno-modulation	Responses	Other observations	First author, journal, year of publication, reference in main text
*In-vitro*	*Aspergillus* conidia and hyphae	Co-culture followed by 2,3-bis-(2-methoxy-4-nitro-5-sulfophenyl)-2H-tetrazolium-5-carboxanilide	Human primary NK cells from PBMC	Negative selection using anti-CD3 microbeads was followed by a positive selection of CD56+CD3- cells using anti-CD56 antibodies. Purity 95%. Viability 98%	E:T 10:1, 20:1, 50:1; 6 hours	1000 U/mL IL-12 every 3rd day during culture	No	Unstimulated and IL-2 stimulated NK cells killed hyphae but not conidia. Hyphae killing range was 15-43% with MOI 10:1-50:1. Killing was better with IL-2 stimulated cells	High level of IFN-γ and GM-CSF produced by NK cells were reduced by *Aspergillus*. IL-2 stimulated cells induced more perforin which aided in hyphae killing	Schmidt J. et al. Infect Dis 2011 (80)
		(XTT) assay								
*In-vitro*	*Paracoccidioides brasiliensis* yeast	Growth inhibition and ^51^Cr release assay against YAC-1 cells for NK activity	Murine splenic cells	Nylon wool non-adherent cells and Percoll fractionation	E:T 50:1 to 500:1; 18 hours	No	No	52-67% growth inhibition was achieved with higher E:T ratios		Jimenez B.E. et al. Infect Immun 1984 (98)
*In-vitro*	*Cryptococcus*	Co-culture of NK cells and fungi followed by Triton-X and enumeration of fungal growth	Studies performed using mostly primary NK YT cell lines and some human NK cells	Human NK cells isolated by magnetic-activated cell sorting (MACS) NK isolation kit (negative selection). Purity >90%.	E:T >100:1; 24 hours	No	No	YT and human NK cells had anti-cryptococcal activity	Perforin was responsible for the anticryptococcal activity of YT cells and primary NK cells. Dependent on PI3K-ERK1/2 signalling. Contact was required between NK cells & fungi	Ma L.L. et al. J Immunol 2004 (103), Wiseman J.C. et al. J Immunol 2007 (104)
*In-vitro*	*Cryptococcus*	Co-culture followed by 3-(4,5-dimethylthiazol-2-yl)-2,5-diphenyltetrazolium bromide (MTT) and fluorescein diacetate viability stain, growth inhibition	Murine	Murine spleen nylon wool non-adherent cells enriched on discontinuous Percoll gradient.	E:T 2:1; 18 hours	No	No	NK cells killed *Cryptococcus neoformans* through effector cell binding and via soluble factors, up to 45% killing.		Nabavi N. et al. Infect Immun. 1985 (99), Hidore M.R. et al. Infect Immun. 1991 (100)
*In-vitro*	*Cryptococcus*	Co-culture	Source from human PBMC	Nylon wool non-adherence and selected by rosetting with sheep erythrocyte/panning.	E:T 40:1 and 200:1; 24 hours	IL-2 1000 U/mL	No	CD16/56 lymphocytes exhibited anti-fungal activity by up to 45%.	CD4 and CD8 lymphocytes also had potent anti-fungal activity. B cells and opsonins were non-essential	Levitz S.M. et al. Infect Immun. 1994 (103)
*In-vitro*	*Candida albicans* yeast	Co-culture followed by XTT assay	Human primary NK cells from PBMC	MACS NK isolation kit (negative selection). Purity > 95%.	E:T 2:1; 4 hours	100 U/mL IL-2, 50 ng/mL IL-15, 1000 U/mL IFN-α, 2000 U/mL IFN-β	No	Primary NK cells induced 20-30% reduction on *C. albicans* viability that was mediated by perforin. Neutrophil anti-fungal activity was also enhanced by NK cells	NK cells phagocytosed *C. albicans.* NK cells degranulate with granzyme B and perforin, and release IFN-γ, GM-CSF,TNF-α in response to *Candida*. Contact dependent activation was shown to be important	Voigt J. et al. J Infect Dis 2014 (77)
*In-vitro*	*Rhizopus oryzae* conidia and hyphae	Co-culture followed by XTT assay for hyphae. For conidia, co-culture with NK cells, followed by lysis and culture enumeration	Human primary NK cells from PBMC	Negative selection using anti-CD3 microbeads was followed by a positive selection of CD56+CD3- cells using anti-CD56 antibodies. Purity 95%. Viability 98%.	E:T/ cell dose not detailed; 6-8 hours	1000 U/mL rhIL-12 every other day during culture	No	Human NK cells exhibited anti-fungal activity against *R. oryzae* hyphae but not conidia.	Perforin was involved in the direct damage of *R. oryzae* hyphae by NK cells	Schmidt S. et al. Immunobiol 2013 (79)
*In-vitro*	*Coccidioides immitis* endospores and spherules	Co-culture of effector cells with *C. immitis* endospores and spherules	Human peripheral blood lymphocytes	Peripheral blood layering, density gradient centrifugation, complementation with Leu-11b, directed against Fc receptor on NK cells	E:T 12.5:1 to 100:1; 4 hours	Interferon (unspecified) 250 U	No	Leu-11b complementation inhibited *C. immitis* killing by 76%	Interferon enhanced peripheral blood leucocyte mediated inhibition of *C. immitis*	Petkus A.F. et al. J Immunol 1987 (101)
*In-vivo* and *in-vitro*	*Aspergillus fumigatus*, pulmonary infection	Adoptive transfer of murine NK cells into mice	Murine splenocytes	Splenocytes were cultured with indomethacin, 2-mercaptoethanol, murine IL-12 and mIL-18 for 5 days. Cultured NK cells then enriched by depletion of CD5+, Ly-6G+, TER-119+, CD22+, and F4/80+ cells. Purity >95%.	2 × 10^6^ cells in 100 μl through tail vein injection	mIL-12 (1 ng/ml) and mIL-18 (100 ng/ml)	Neutrophil depletion	Neutrophil depleted mice which received wild type NK cells had approximately 80% lower lung fungal chitin content	MCP-1/CCL2 mediated influx of lung NK cells through CCR2	Morrison B.E. et al. J Clin Invest. 2003 (91)
*In-vivo* and *in-vitro*	*Aspergillus fumigatus*, pulmonary infection	Adoptive transfer of murine NK cells into mice	Murine splenocytes	Splenocytes were cultured with murine IL-12 and mIL-18 for 5 days and negatively selected by depletion of CD5+, Ly-6G+, TER-119+, CD22+, and F4/80+ cells. Purity >95%.	2 × 10^6^ cells in 100 μl through tail vein injection; 3 days	mIL-12 (1 ng/ml) and mIL-18 (100 ng/ml)	Neutrophil depletion	Depletion of NK cells led to a 2-3 fold increase in lung fungal chitin. NK cell adoptive transfer resulted in at least 2 fold reduction in lung fungal burden.	In neutropenic mice NK cell-derived IFN-γ was shown to be important in defending against *A. fumigatus* through CXCL9 and CXCL10	Park S.J. et al. J Immunol. 2009 (92)
*In-vivo* and *in-vitro*	*Aspergillus fumigatus*, pulmonary infection	Adoptive transfer of human NK cells into mice	Human PBMC	Human PBMC were expanded and activated by co-culture with K562 cells genetically modified to express 4-1BB ligand and membrane-bound IL-15 with CD3 depletion. Purity and viability >92%.	E:T of 2:1 or 1 × 10^7^ cells in 200 μl; 4 days	10 U/mL rIL-2 over 1 week	Cortisone acetate (250 mg/kg/200 µL) and cyclophosphamide (250 mg/kg/100 µL)	Mice treated with the expanded NK cells had significantly lower fungal burden (40% reduction) in the lungs when compared to untreated mice	Highest efficacy was observed against *A. fumigatus* conidia mediated by dectin-1 receptor on NK cells which led to augmented release of perforin, that resulted in direct cytolysis	Soe W.M. et al. J Fungi (Basel) 2020 (83)

The advent of the magnetic-activated cell sorting (MACS) platform has also revolutionized our capacity to sort cellular subtypes *via* positive or negative selection, enabling us to select for CD56+CD3- NK cells with more than 90% purity. This platform has been widely employed to yield enriched NK cells for further *in vitro*, and *in vivo* anti-fungal studies, focusing on more difficult-to-treat moulds such as *Aspergillus* and *Zygomycetes*. Interest also remains in the treatment of pathogenic yeasts *Candida* and *Cryptococcus*. In tandem, a deeper mechanistic understanding of the anti-fungal properties of NK cells were also gained. For instance, the major roles played by cytokines such as IFNγ; NK-released perforins; the requirement for physical contact between effector-target cells; and, a predilection for specific pathways (e.g. PI3K-ERK1/2 signaling in NK-mediated anti-*Cryptococcal* activity) (104, 105). The use of human NK cells purified from peripheral blood also regularly involved priming cytokines IL-2, IL-12, IL-15 and IFNα/β concoctions which enhanced NK cellular activity. Against *Aspergillus*, human NK cells were able to kill *A. fumigatus* hyphae, but not resting conidia *in vitro*. Hyphae killing, measured *via* methoxynitrosulfophenyl-tetrazolium carboxanilide (XTT) assays ranged between 15% to 43% measured hyphae killing of 15-43% was achieved, but required high E:T ratios of 10:1 to 50:1 (80). Similar results were seen against *Zygomycete*s, whereby *R. oryzae* hyphae (but not conidia) were susceptible to NK killing (79), as well as in *C. albicans* (77).

Against the background of the above *in-vitro* study outcomes saw the development of adoptive transfer of NK cellular therapy against IFI in animals. This was first successfully demonstrated in 2003 with murine splenocytes cultured in IL-12 and IL-18 and purified through negative selection with 95% yield. Two million NK cells injected into immunocompromised mice in a pulmonary aspergillosis mice model *in vivo* saw significant reduction in lung fungal burden (91, 92). The most recent attempt involved the use of adoptive xeno-transfer of human NK cells to treat mice with pulmonary aspergillosis. Of note, these human NK cells were highly activated, expanded by co-culture with K562 cells genetically modified to express 4-1BB ligand and membrane-bound IL15 (K562-41BBL-mbIL-15). Promisingly, cytotoxicity was observed with a significantly lower E:T ratio of 2:1 where the infusion of 10 million cells led to an approximately 40% reduction in fungal growth, independent of anti-fungal drugs (83).

## State of art: Clinical applications and considerations for NK Cell therapy from cancer to IFI

Much of what is currently known about the clinical application of NK cells stems from decades of cancer research in the use of NK cell infusions for cancer treatment (47). NK cells can be obtained from peripheral blood of allogeneic donors, differentiated umbilical cord blood, induced pluripotent stem cells (iPSCs), or irradiated cells of the NK-92 cell line.

The clinical preparation of NK cells for cancer therapy depends on the source of NK cells and it can be broadly divided into the following processes: 1) harvesting from source; 2) *ex vivo* NK cell preparation which may require differentiation for stem cell sources, and usually involves expansion to generate higher numbers of effector cells, 3) patient lymphodepletion chemotherapy prior to infusion; and, 4) patient IL-2 therapy after infusion. In this section, we will further discuss what is currently known about the clinical application of NK cells in human clinical trials.

## Sources of NK cells

### Peripheral blood NK cells

**Figure 1:**
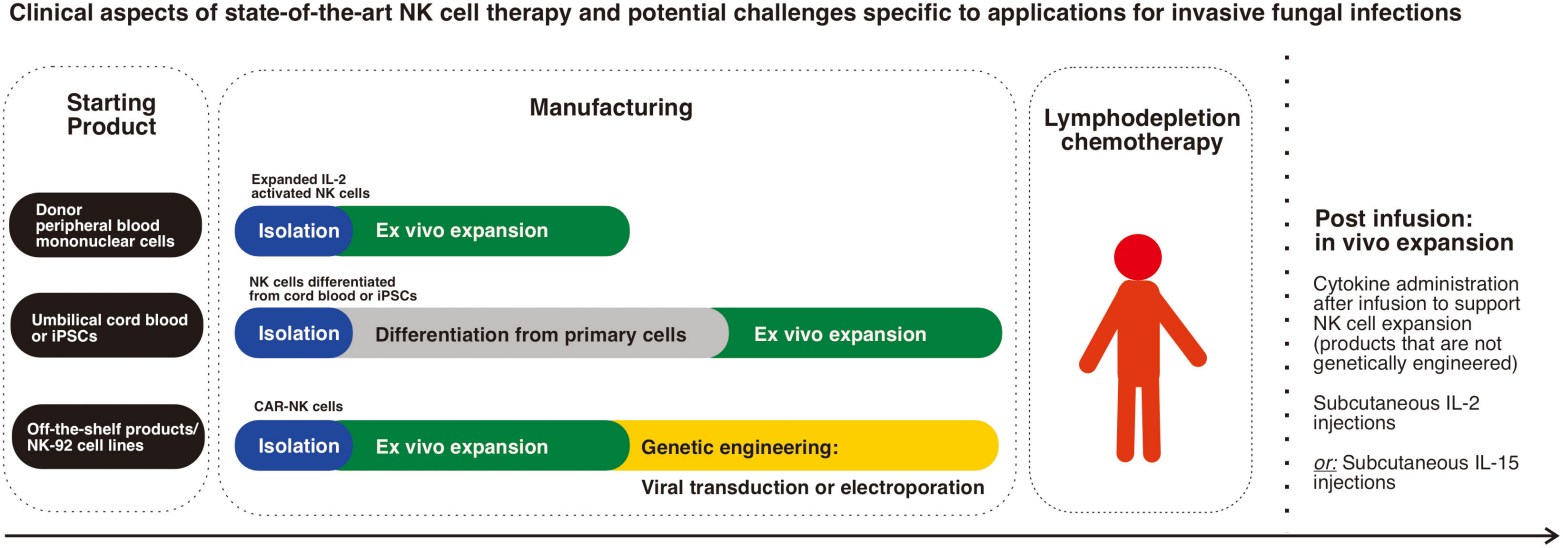
Clinical aspects of state-of-the-art NK cell therapy and potential challenges specific to applications for invasive fungal infections: The main sources of NK cells in clinical trials are donor peripheral blood mononuclear cells which are the most commonly used, while next generation genetically modified products using stem cells (umbilical cord blood cells or iPSCs) are progressively emerging. Schematic diagram representing the main steps in cell manufacturing highlights the complexity and time needed to produce stem cell differentiated products in addition to the need for genetic engineering of CAR-NK cells. The need for lymphodepletion chemotherapy and post infusion cytokine administration, which is the case for most non-genetically engineered products is an important clinical aspect of NK cell therapy.

The peripheral blood is the most common source of NK cells in clinical trials (**Figure 1**). Allogeneic donors of peripheral blood NK cells have been preferred over autologous NK sources in the setting of anti-tumour activity for patients with cancer (47). This is primarily due to the self-HLA expression on tumour cells that inhibit the activity of autologous NK cells (47). HLA typing is also required to identify suitable donors. Although allogeneic NK cells generally do not cause graft versus host disease (GVHD) directly (106, 107), adequate T-cell depletion procedures (typical safety limit: 5 x 10^4^ per kg body weight) are still required (108, 109). 

The process of isolation and enrichment of NK cells from peripheral blood mononuclear cells starts with a leukapheresis procedure (110). In this process, white blood cells are removed from the blood of the donor while the remaining blood components are returned *via* an apheresis machine (111). Mononuclear cells are further enriched by centrifugation with Ficoll density gradient centrifugation before T cell depletion with CD3 immunomagnetic beads, followed on with CD56 enrichment (112).

Because peripheral blood NK cells of healthy individuals are usually in a resting state, pre-activation is necessary to optimize functionality using IL-2 or IL-15 (113). Short term exposure to high dose IL-2 for 12-16 hours at doses of 1000 international units per millilitre has been shown to adequately activate NK cells in the study by Miller et al. (107). IL-15 has been described to maintain NK cell cytotoxicity for a longer period than IL-2 without repeat exposure (114, 115), however other studies have suggested the possibility of cell exhaustion with other IL-15 dosing regimens (116). Other combinations involving 12-16 hour incubations with IL-12, IL-15 and IL-18 have also been used in clinical trials as pre-activation prior to infusion in patients (117). Recently, inhibitors of glycogen synthase kinase 3 (GSK3) have also been described to induce NK cell maturation and cytotoxicity (118).

### Differentiation from stem cells: Umbilical cord blood and iPSCs

NK cells can also be obtained through the differentiation of stem cells such as umbilical cord stem cells and iPSCs. Umbilical cord banks allow the advantage of selection of donors with a specific HLA or NK receptor profile. A higher number of naïve NK cells exist in cord blood (approximately 30%) than in peripheral blood (approximately 10%) (119). While they have lower spontaneous killing of target cells due to lower expression of perforin and granzyme B, they express the same amount of activating receptors (120). With *ex vivo* activation and expansion, lower cytotoxicity can be overcome to allow broader off-the-shelf applications, including CAR-based therapy (121), given the ubiquity of cord blood banks.

CD34+ hematopoietic cells from umbilical cord blood can be differentiated into NK cells with the use of stem cell factor (SCF), IL-2, IL-7, IL-15, and other growth factors (122, 123). Haematopoietic differentiation of iPSCs can be first induced with SCF, vascular endothelial growth factor (VEGF) and bone morphogenetic protein 4 (BMP4) (124). Thereafter, NK cell differentiation is stimulated using IL-3, IL-7, IL-15, SCF and FLT3L (125). These differentiated NK cells can then be expanded by co-culture with K562 cells with membrane bound IL-21 (125, 126). However, the main limitation of differentiated cells as a source is the significantly longer duration of culture required (about 7 weeks) (127) which may not be practical for clinical use in patients with rapidly progressing conditions.

### Off-the-shelf genetically modified NK-92 products

Emerging data suggest the potential for NK-92 cells to be an off-the-shelf source of NK cells for further engineering and clinical development. First established in 1994, NK-92 is a cell line derived from the peripheral blood of a patient with non-Hodgkin’s lymphoma that was first established in 1994 (128); it and has been found to have a high expression of activating receptors (129), granzyme B and perforin (130).

The expansion of NK-92 cells has been adapted to good manufacturing practice (GMP) conditions with a doubling time of 24 to 36 hours with IL-2 support (131, 132). Phase I clinical trials using NK-92 cells in patients with hematologic malignancies and solid tumours have demonstrated safety with some clinical response (133–136). Genetic modification of NK-92 cells through viral transduction with CARs against a variety of cancer targets such as CD19 and CD20 for B-cell malignancies (137–139) have also been developed. The first CAR NK-92 clinical trials were conducted in patients with relapsed or refractory acute myeloid leukemia, using a lentiviral transduced third-generation CAR targeting CD33 (140). While clinical efficacy was not observed, these early studies demonstrated safety as no grade 3-4 adverse events were observed.

While NK-92 cells present an attractive, off-the-shelf option for NK cell-based therapy, a potential major drawback is limited persistence given that the cells are irradiated. Furthermore, most protocols do not include lymphodepleting chemotherapy which limits persistence as the cells are more likely to be rejected very quickly.

## Ex-vivo preparation of NK cells: expansion through co-culture with K562 cells

NK cell activation and proliferation can be induced through co-culture with the chronic myelogenous leukaemia derived cell line, K562 (141). Transduction of K562 with both mbIL-15 and 4-1BBL (14, 46, 113) which are then irradiated, further improves NK cell proliferation following co-culture, a method which has also been adapted to clinical use in large-scale, GMP conditions. While irradiation restricts any proliferation of K562 cells, NK cells also effectively kill K562 cells;, ensuring that there will be no viable K562 cells infused to patients (110).

GMP cultures of NK cells isolated from peripheral blood mononuclear cells lasting 10 days can achieve more than 300-fold NK cell expansion (46, 110, 113). This allows sufficient cells for multiple infusions from a single leukapheresis product (110, 142). An even higher amount of NK cells can be generated by prolonging the duration of culture, or through the addition of more irradiated K562-mbIL-15-4-1BBL cells for up to 8-15 weeks, following which senescence may start to set in (14).

Other modifications to K562 cells in an attempt to improve NK cell expansion after co culture have also been studied: 4-1BBL with IL-15 receptor alpha chain (109); 4-1BBL with IL-21 (143); CD64, CD86, CD19 with 4-1BBL; mbIL-15, mbIL-21 or both (111, 126, 144); OX40 ligand with IL-2, IL-15 and IL-21 (145).

## Optimizing the host for NK cell therapy: lymphodepletion and cytokine administration

Allogeneic NK cells only persist for 2-3 weeks after infusion. Lymphodepletion is primarily used to improve persistence by overcoming rejection of NK cells by the recipient’s immune system (69, 110, 146). Fludarabine and cyclophosphamide are the most widely used drugs to preferentially deplete recipient lymphoid cells prior to NK cell infusion (107). In the pioneering work by Miller et al. (107), deeper immunosuppression through the addition of cyclophosphamide at 60mg/kg for 2 days to a fludarabine regimen of 25mg/m2 for 5 days led to higher levels of serum IL-15 and resulted in better engraftment. With these lymphodepletion regimens, allogeneic NK cells typically persist for 14-21 days following infusion (69, 110, 146, 147), including that of umbilical cord blood derived NK cells (148).

In addition to lymphodepletion, IL-2 is also commonly administered to improve survival and expansion of donor NK cells. Typically, 6 doses of IL-2 are given subcutaneously over 2 weeks after NK cell infusion (110, 127, 146, 149, 150).

In a study by Miller et al. (107), haploidentical IL-2 activated NK cells were observed to have increased NK cell expansion with higher serum IL-15 levels in patients who received lymphodepletion plus IL-2-diptheria toxin fusion protein, as compared to those who received lymphodepletion chemotherapy alone (127).

IL-15 and its variants have also been used to support NK cell expansion in clinical trials. Cooley et al (151) showed that NK cell expansion was higher than what had been previously described with IL-2; however, subcutaneous IL-15 injections were associated with cytokine release syndrome and associated neurotoxicity. Increased toxicity was hypothesized to be due to IL-15 stimulation of monocytes and T cells in contrast to actual NK cell activation (151).

## Adaptation of current NK cellular technology to treatment of IFI: identified challenges and modifications

The perceived attractiveness of NK cellular therapy lies in the capacity of NK cells to directly kill target cells and stimulate the host immune response, without need for prior antigen-specific sensitization, with minimal risk of inducing GVHD (152). While *in vitro* and animal *in vivo* NK studies against fungi to date seem promising, these are rudimentary first steps in using NK cells with anti-microbial intent against pathogens compared to the experience accumulated to date with NK infusions for their anti-tumour activity, from which challenges have been recognized. For instance, the requirement for continued IL-2 administration post- NK cell infusion has been pivotal in sustaining viability and expansion but its dosing is limited by the adverse IL-2 cytokine-linked side effects experienced by patients like malaise, nausea, vomiting (153). The potential merits of the use of higher doses of IL-2 (up to 6,000 IU) (113) or with addition of IL-15 and IL-12 have since come under question. IL-15 enhances cytotoxicity, but also increases the risk of immune exhaustion (116). More critical cytokine-related toxicities such as worsening leucopenia, granulocytopenia and thrombocytopenia are likely already inherent in patients susceptible to invasive fungal infections, hypotension, and capillary leak syndrome. The development of IL-15 analogues, such as N-803, primes NK cells to produce much needed IFNγ and TNFα (154) against the fungi. NK cell expansion using irradiated K562 cells with membrane bound IL-15 and 4-1BBL is now being used to produce a good yield of expanded NK cells for cellular therapeutics.

While anti-tumour treatment largely involves single infusions of NK cells, the optimal number of infusions of NK cells, which has important implications on its applicability, remains unknown. The duration of viability of a single infusion of NK cells is estimated to be between 2-3 weeks, subject to rejection by the recipient’s immune system. In order to allow the infused NK cells to dwell and exert their anti-fungal effect, lymphodepletion chemotherapy also acts as a double-edged sword since they are also well recognized to compromise even an immunocompetent host’s immune mechanisms. In the setting of heavily pre-treated patients, or those with existing co-morbidities, fitness to undergo lymphodepletion is a major consideration, given the dose intensity required. In fact, IFI patients who are post stem cell transplant (SCT) or on intensive chemotherapy, are already lymphodepleted by nature of their therapy. Hence, one might view the use of NK adoptive therapy from an allogenic source (or ideally, from the SCT donor) as befitting, under such circumstances.

Historically, the use of autologous NK cells for anti-tumour intent has not yielded significant efficacy. This has been attributed to HLA signals on the tumour cells which may inhibit autologous NK cells from activating and effecting cytotoxicity. However, this is unlikely to be of concern against an invading fungus; instead, it could potentially be an alternative source of NK cells in addition to a conventional allogenic option.

As with other more established forms of personalized cell-based therapy such as CAR-T cell therapy, the issues of manufacturing such as: quality of starting material, turnaround time, schedule availability and requirement for quality control testing prior to product release, need to be addressed when considering the practical aspects of clinical NK cell therapy. Given the clinical urgency, an autologous source may be the most practical. However, if a patient is already in a dire state where the invasive fungal infection is in fact an effect of underlying profound immunosuppression, it may render the harvesting of a suitable starting material impossible. In the same vein, the need for HLA matching and the search for an allogeneic healthy donor starting product may take more time than what a critically ill patient may have.

The role of NK cells as innate lymphocytes effecting non-discriminatory, anti-tumour, and possibly anti-microbial, activity is a double-edged sword. NK cells utilize inhibitory receptors like KIRs which interact with MHC-1 to distinguish between ‘self’ and ‘non-self’, marking out the latter for effector response. While they are not known to display clonotypic receptors, what is apparent is that NK preparation and priming seems to have an effect on fungal morpho-typic activity. NK cells primed by IL-2 by Schmidt et al. displayed specificity for *A. fumigatus* and *R. oryzae* hyphae but not conidia (80). Conversely, expanded NK cells expressing K562-41BBL-mbIL-15 by Soe et al. have been observed to be most efficacious against *A. fumigatus* germinating conidia, and this was attributed to the Dectin-1 receptor (83). As a therapeutic benchmark, voriconazole effectively targets both germinating conidia and active growing hyphae (155). Given the morpho-typic predilection against fungi that has been observed with NK cells from different sources, further development of NK cells with enhanced activity against both conidia and hyphae would be desirable.

## The goal ahead: augmenting NK anti-fungal activity through genetic engineering

Efforts in engineering NK cells to augment function have been made *via* viral transduction or electroporation of mRNA (110, 156). NK cell proliferation is critical in allowing DNA integration of the gene of interest, especially when retroviral transduction is used (46). Gene expression is also generally improved in lentiviral transduction if the cells are proliferating (157). Given concerns regarding oncogenic mutagenesis with viral transductions, mRNA electroporation – a method that does not involve viral vectors – has also been studied. Despite being less costly and faster to prepare, a major limitation is that gene expression is transient and is generally lost within a week (156). While this may be a limitation for cancer-related applications, short-term expression may be still be acceptable for other indications beyond oncology.

To date, genetic engineering efforts have been geared towards 1) enhancing activation and proliferation, 2) decreasing inhibition, and 3) redirecting cells toward specific targets. While most of the studies have been conducted in the context of oncology, significant parallels may also be applicable to an anti-fungal setting.

**Figure 2:**
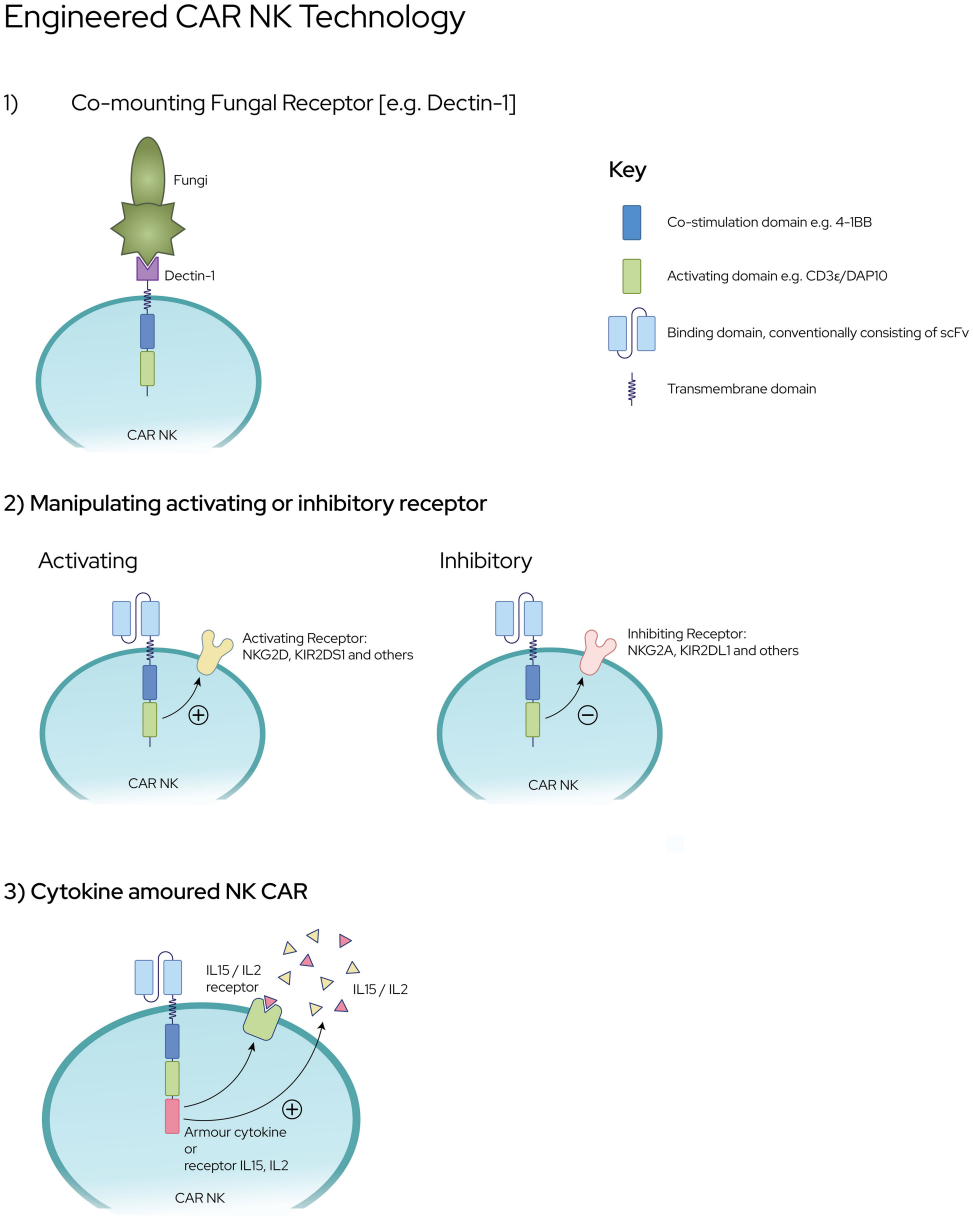
Engineering CAR NK Platforms against Invasive Fungal Infections [1] CAR NK cells co-expressing fungal specific receptors e.g., Dectin-1 fused to co-stimulation and activating domains [2] Enhanced expression of NK-activating receptors or down regulation of NK-inhibitory receptors upon ligation [3] NK cells with ‘cytokine-armouring’ capability for superior persistence and sustenance against fungal pathogens.

CAR engineering of NK cells is an emerging field that leverages on the success of CAR-T cell technology in cancer [**Figure 2**]. The potential major advantage of CAR-NK cells over CAR-T cells is its potential for off-the-shelf use, given its HLA-unrestricted killing (158), which abrogates the need for complex testing and personalised manufacturing, in often dire and urgent clinical scenarios. CAR-NK cells have also demonstrated extended *in vivo* persistence and a favourable safety profile in ongoing clinical trials (159). Primarily designed to re-direct T cells towards specific targets such as CD19 expressed on B-cell malignancies, second generation CAR-T cells first demonstrated cytotoxicity through the incorporation of the 4-1BB co-stimulatory domain (46). Further observations of a higher basal level of activation that was not dependent on antigen binding have been characterised in a phenomenon known as tonic signalling (160, 161). This suggests that CAR constructs not only redirect T cells to specific targets, they also interact with endogenous receptors leading to more T cell activation (162). While the effects of tonic signalling have not been elucidated in CAR-NK cells, similar cooperative activation in NK cells are plausible (158). With the right target antigen, CAR-NK cells directed against fungi have significant potential for further clinical development.

Dectin-1, also known as CLEC7A, is a C type lectin receptor which recognizes beta-glucan, a ubiquitous polymer in the cell walls of fungi such as *Aspergillus*, *Candida* and *Zygomycetes* (163, 164). Beta-glucan is most abundantly expressed on actively growing fungal morphotypes such as germinating conidia and hyphal tips (165, 166). Hence, the generation of CAR-NK cells co-expressing Dectin-1 receptor may literally ‘nip the problem in the bud’ given its enhanced specificity over dormant or inactive fungal conidia. In an attempt to study this, Dectin-CAR-T was innovatively modified through the fusion of Dectin-1 receptor to CAR cassette encoding chimeric CD28 and CD3-ζ (167). These Dectin-CAR-T, through their specificity for beta-glucan, were able to induce hyphal damage, thereby functioning as treatment against aspergillosis in mice. This proof-of-concept capability harbours the potential to engineer NK cells with similar intent whereby the likelihood of rejection is lower than T lymphocytes.

Next, is the possibility of manipulating the balance between NK activating receptors (e.g., NKG2D-DAP10) and inhibitory receptors (e.g., NKG2A and KIR) through genetic engineering involving CRISPR-Cas9 gene editing technology. This has now been made possible with the success of CAR-T technology which can also be applied to NK cells as CAR-NK cells with specific targets and increased cytotoxicity. Engineered CAR-NK cells with membrane bound IL-15 (168) (or, with 4-1BBL) and enhanced NKG2D–CD3ζ–DAP10 expression promise to exert greater cytotoxicity with increased cytokine production and degranulation (169). Conversely, down regulation of NK inhibitory receptors is now possible through the use of (i) gene editing techniques; (ii) single-chain variable fragments targeting NKG2A protein expression at the endoplasmic reticulum, and (iii) anti-KIR antibodies (170). Intuitively modifying NK cells to augment expression of IL2R and IL15R also serves to increase the sensitivity of NK cells to enrichment and expansion.

Cytokine “armoured” NK cells are being developed through genetic CAR engineering of NK cells. On the basis that continuous cytokine stimulation of NK cells during *ex vivo* NK cell preparation renders the cells ‘cytokine-addicted’, these cells are being developed to reduce the effects of “cytokine addiction” such as decreased persistence *in vivo* (171). Cytokine armouring is being developed in the forms of 1) soluble cytokines that are secreted which also activate other immune cells (172) and, 2) membrane-bound cytokines that are triggered upon cellular interactions (173). When compared with CAR alone, IL-15-armoured CAR NK cells demonstrated superior persistence (168), not only *in vivo* in preclinical models but also in a clinical study of patients with CD19 CAR-NK cells (159). A similar approach can also be applied in the setting of anti-fungal NK cell therapy to augment NK cell function and overcome the challenges of an immune milieu that is often low in IFNγ and TNFα in the setting of an invasive fungal infection.

The ultimate objectives in optimizing NK cellular therapy against fungi lie in enhancing activation and expansion of NK cells to an ideal E:T ratio. This translates to good cell numbers, viability, and enhanced activity. Beyond the traditional ‘single donor’, use of hematopoietic stem and progenitor cells (HSCs) and iPSC lines (174, 175) provide the advantage of an accessible bank of matched donors. However, inherent obstacles such as the complex cell manufacturing process, need to be overcome through development of off-the-shelf products that do not require HLA matching, given the often-urgent clinical scenarios. Stem cell products can also potentially acquire significant new mutations (176) during the differentiation process which presents an added challenge of regulatory concerns about potential oncogenicity. Other off-the-shelf options such as genetically modified NK-92 cells are promising in terms of time-to-infusion advantage; however, the cytotoxicity and persistence of NK-92 cells that have been lethally irradiated (177) remain to be established until further clinical data from ongoing trials become available (178).

Further studies need to be directed toward an ideal setting where 1) the starting material is readily available; 2) manufacturing can be completed in a reasonable time frame, if not, an off-the-shelf product; 3) lymphodepletion is not required (especially in already immunosuppressed patients); and, 4) NK cells are able to expand *in vivo* and maintain their own activating signals to mediate effective cytotoxicity.

## Conclusion

NK cell technology has improved by leaps and bounds in recent decades, making adoptive cellular transfer a viable therapeutic option that can now be considered against difficult-to-treat fungal infections. However, it is to be highlighted that the use of NK cells as antimicrobial, anti-fungal therapy is unlikely to be implemented independently as a form of monotherapy. A practical strategy would be to administer NK cellular therapy in conjunction with conventional therapeutics such as the new generation azole anti-fungal drugs (i.e., voriconazole, posaconazole, isavuconazole) or even liposomal amphotericin B. The basis for supporting the deployment of a targeted immune- augmenting therapy alongside conventional antimicrobials is strong, given that many patients with invasive fungal infections are immunocompromised.

## Author contributions

All authors listed have made a substantial, direct, and intellectual contribution to the work and approved it for publication.
